# Action feedback affects the perception of action-related objects beyond actual action success

**DOI:** 10.3389/fpsyg.2014.00017

**Published:** 2014-01-24

**Authors:** Wladimir Kirsch, Elisabeth Königstein, Wilfried Kunde

**Affiliations:** Department of Psychology, University of WürzburgWürzburg, Germany

**Keywords:** visual perception, action, knowledge of results, action success, action ability, perception-action coupling

## Abstract

Successful object-oriented action typically increases the perceived size of aimed target objects. This phenomenon has been assumed to reflect an impact of an actor's current action ability on visual perception. The actual action ability and the explicit knowledge of action outcome, however, were confounded in previous studies. The present experiments aimed at disentangling these two factors. Participants repeatedly tried to hit a circular target varying in size with a stylus movement under restricted feedback conditions. After each movement they were explicitly informed about the success in hitting the target and were then asked to judge target size. The explicit feedback regarding movement success was manipulated orthogonally to actual movement success. The results of three experiments indicated the participants' bias to judge relatively small targets as larger and relatively large targets as smaller after explicit feedback of failure than after explicit feedback of success. This pattern was independent of the actual motor performance, suggesting that the actors' evaluations of motor actions may bias perception of target objects in itself.

## Introduction

How we perceive objects depends on the quality and success of motor actions aimed at these objects (Wesp et al., 2004; Witt and Proffitt, 2005; Witt et al., 2008; Cañal-Bruland and van der Kamp, 2009; Witt and Dorsch, 2009). When an actor intends hitting an object, this object is perceived larger the more successful s/he was. For example, successful golfers judge golf holes as larger than less successful golfers (Witt et al., 2008), or football players who hit goals often see goal post wider apart than players who hit the goal seldom (Witt and Dorsch, 2009). Apparently, visual information is enriched by motor-related variables which then jointly form visual experience (cf. Proffitt and Linkenauger, 2013). Such motor variables were assumed to relate to some aspects of the actors' actual ability to act (Witt, 2011), such as to his/her “momentary form” (Lee et al., 2012) or the “variance of performance” (Proffitt and Linkenauger, 2013).

When considering motor influences on perception it is important to distinguish two aspects of motor actions, namely the *actual* action result and the actor's *knowledge* of that result. The actual result denotes the outcome of the action in terms of objective measures, such as whether a target object was hit or to which extent it was missed. Knowledge of result, by contrast, denotes what the actor knows or believes about that result. Often both types of information are redundant. When a golfer tries to put a ball, s/he most often directly sees or hears whether the put was successful or not. But there are also situations where actual and known results dissociate. Trying to put a golf ball into a hole behind a hill or from a sand bunker would be an example. Whether the ball actually hit the target becomes apparent only after one has climbed on the hill or out of the bunker and a well-meaning friend or an external force like a wind gust might turn an actual miss into an experienced hit meanwhile. Comparable experimental tasks with variable amounts of knowledge of result have been studied extensively in the motor learning literature (Salmoni et al., 1984). The dissociation between actual and known or believed result becomes even more obvious when it comes to evaluate an objective action outcome against a certain standard. For example, whether six hits out of ten shots from the basketball free throw line is a good or bad performance depends on what the actor knows or believes about the average performance of other players or his/her own performance in previous shots.

The present study explored if evaluation of action outcome associated with the knowledge of result has an impact on the perception of task-related objects beyond the actual action ability. Previous observations already lend support to a role of knowledge of results on perception indirectly. Specifically, influences of action on perception are typically observed in judgments measured after motor performance, hence after knowledge of result had become available (but see Lee et al., 2012 for an exception). Consequently, good performers may see a target not different as poor performers, but they may estimate target size as larger just because they experienced to be successful briefly before (cf. Cooper et al., 2012, p. 236; Wesp et al., 2004, p. 1265). In other words, action evaluation processes rather than action ability *per se* might be responsible for many previously reported observations^1^. Such an outcome dependent perceptual modulation may be useful for preparation of future actions: seeing (or remembering) a target as smaller after a miss, e.g., may encourage the actor to exert additional resources (cf. Witt and Dorsch, 2009).

Finding that knowledge of action result affects perception in a similar way as can be expected based on explanations related to actor's action ability (i.e., larger estimates after more successful actions, see above) would suggest that it is not action ability *per se* that biases perception but instead what actors believe about their ability, including all emotional and motivational consequences that come about with such believes. From a methodological point of view, such a finding would point to the necessity of controlling for knowledge of result in future studies of action influences on perception.

**Figure 1:**
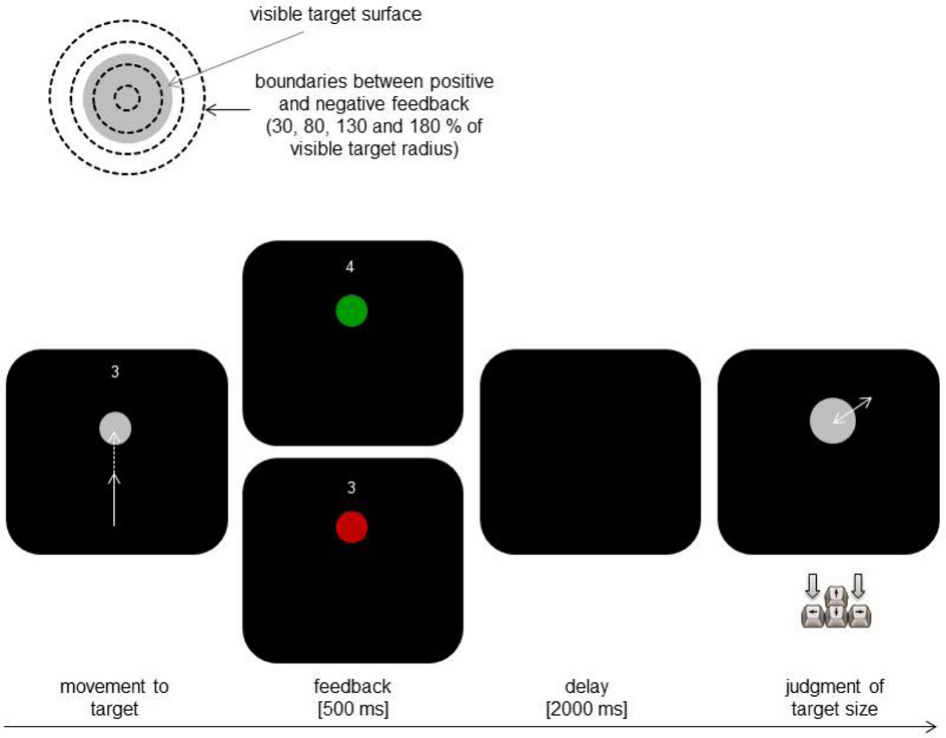
**Schematic illustration of the critical manipulation (upper part) and of the main trial events in Experiment 1 (lower part)**.

In Experiment 1, we employed a simple aiming task to test for such influences. Participants were asked to repeatedly hit circular targets of varying diameter by pointing movements. Visual feedback was restricted to the start of the movement so that the actual success of the action (whether the target was hit or not) was not directly discernible. Instead, knowledge of result (hit or miss) was provided at the end of the movement. Importantly, the spatial area counting as a hit varied unpredictably and unbeknown to the participants. In some trials the accepted hit area was smaller than the (real) target area, whereas it was larger than the target area in other trials (see Figure 1, upper part). This manipulation not only created cases in which participants received valid feedback, but also cases in which participants actually hit the target but received the feedback that they did not, and conversely, cases in which they actually missed the target but received feedback of a hit. In other words, we deconfounded actual action ability from knowledge of result. After being provided with knowledge of result, participants judged the size of the target. The main question of interest was whether size judgments after apparent hits differ from judgments after apparent misses.

Experiments 2 and 3 primarily served as control experiments in which we aimed to replicate the pattern of results observed in Experiment 1 under slightly different stimulus conditions. In Experiment 2, the target was surrounded by either smaller or larger context stimuli to induce the Ebbinghaus illusion. This allowed us to test whether the employed method of adjustment is sensitive to changes in apparent target size. Demonstrating such an effect would indicate that changes in judgment behavior following feedback manipulations are due to subjective changes in perceived target size rather than due to other task specific factors relating to the used judgment procedure or stimuli (such as due to differences in pressing buttons during the judgment). Moreover, we were interested in whether a performer is more likely to hit the target that is seen to be bigger (Witt et al., 2012). It has been proposed that if a target appears bigger the perceiver may feel more confident during the action and thus, may enhance his/her performance (because s/he might expect to be more able to hit the target that appears to be easier to hit, cf. Witt et al., 2012). Accordingly, we assumed that if action ability and size perception were directly linked to each other, an increase in apparent size should result in an increase of ability to hit the target (i.e., in a general increase of hit rates). Finding such an effect would, of course, not rule out that action evaluation plays a role in size estimation. It would rather suggest that knowledge of results might be one of several other factors mediating between perceptual changes and motor behavior.

Experiment 3 aimed at testing whether the kind of presented feedback was responsible for the results observed in Experiment 1. In Experiment 1, the target got temporary green after a hit whereas it got red after a miss before size judgments were performed. Thus, target color rather than experienced movement success may have an impact on size estimates and accordingly we changed the assignment of the colors to the feedback conditions.

Note that the main purpose of this study was not to test the impact of actual action ability on perceived target size *per se*. Instead we wanted to test whether knowledge of results has an impact beyond action ability. To do so, we employed a regression approach on the individual trial level in addition to the standard Analysis of Variance (ANOVA) approach on aggregated data (cf. Notebaert and Verguts, 2007). The tenet of this approach is to test whether there remains an impact of knowledge of results on individual perceived target size, after influences of actual movement performance on perceived target size have been analytically removed.

## Experiment 1

If the previously reported relationship between motor performance and perceptual judgments (i.e., higher performance leads to larger estimates of action goals) is exclusively due to action ability, then judgments of target size after an experienced hit should not differ from judgments after an experienced miss when real motor performance is held constant. In contrast, if the relation between motor performance and perceptual judgments is primarily due to the processing of action feedback then targets should be judged as larger after an apparent hit than after an apparent miss, independent of real motor performance. Another pattern of systematics in judgments depending on action outcome would indicate that perceptual estimates measured after an action do not exclusively reflect action abilities but are also the result of action evaluation processes. This would point to the necessity of experimental setups that control for these often confounded variables.

### Methods

#### Participants

Twenty-three participants participated. They gave informed consent and received monetary compensation or course credit for their participation. One participant had a not corrected visual impairment. Her data was excluded from analyses. The final sample included 15 females and seven males [mean age (*SD*) = 28 (9) years, ranging from 20 to 53 years of age; two participants reported to be left handed].

#### Apparatus

The experimental apparatus consisted of a digitizing tablet, a digitizing stylus, a monitor and a semi-silvered mirror (see Figure 1 in Kirsch et al. (2012). A digitizing tablet (Wacom Intuos 2 A4) was placed on a table. A monitor was mounted ~48 cm above the tablet. A semi-silvered mirror was placed in the middle between the monitor and the tablet. By means of the monitor, virtual images could be projected on the plane of the mirror that prevented the vision of the arm. The lab was dimmed during the experiment. One pixel (px) of the monitor measured approximately 0.38 mm on the screen. The relation between the stimulus position indicating the position of the stylus and the actual position of the stylus was aligned so that the visible feedback corresponded approximately to the actual stylus position (i.e., we did not manipulate visual feedback during movement execution).

#### Procedure and design

Participants sat in front of the apparatus and were asked to lean their forehead on an upper part of the apparatus. Size estimations were performed with the left hand whereas the stylus was moved with the right hand.

Figure 1 illustrates the main trial procedure. At the beginning of each trial participants moved the stylus to the start position presented as a blue dot of 4 px in size in the middle lower part of the display. The actual position of the stylus was displayed by a gray dot (4 px). Reaching the start position triggered the presentation of a gray circular target above the start position and of an additional short text asking to initiate the next attempt to hit the target (bottom left). The distance between the center of the target and the start position was always 275 px. The target was always in front of the start position and the positions of the target center and of the starting point did not change during the experiment (i.e., the direction of movements was kept constant). The visual feedback of the current stylus position disappeared after a half of the target distance was covered (i.e., after the distance between the y-coordinate of the starting point and that of the current stylus position exceeded the first half of the distance between the starting point and the target). Finishing the movement was to be confirmed by pressing a stylus button. The target changed its color for 500 ms from gray to green in case of a hit (i.e., the endpoint of the movement was within the experimentally manipulated target area; see below). The target color turned from gray to red in case of a miss. The current number of hits achieved in the present block was continuously presented above the target during the initial phase of a trial (cf. Figure 1).

Following a delay of 2 s with a blank screen, participants were to reproduce the size of the target using a method of adjustment. A gray circle was presented at the position of the target. Additionally, a short text asking to start the judgment was displayed on the bottom left. The initial radius of this circle measured either a half or one and a half of the true target radius^2^. Participants were required to adjust the size of the circle to the size of the previously seen target. Pressing the right arrow key on a keyboard (discrete as well as continuous) increased the size and pressing the left arrow key decreased the size. The estimation was confirmed by pressing the enter key. If the initial circle size was not changed before the enter key was pressed, error feedback was presented and the judgment procedure was repeated.

Following this judgment procedure, a short text asked the participant to move the stylus to the start position and the blue dot indicating the start position appeared. Also, after a half of the previous target distance was passed, the gray dot indicating the actual stylus position re-appeared.

The critical manipulation was related to the size of the area separating the feedback of a hit from the feedback of a miss (henceforth referred to as “feedback factor”). Participants received positive feedback when the endpoints of their movements fell into an imaginary circle with a radius of 0.3, 0.8, 1.3, or 1.8 of the visible target radius. In other words, the feedback of movement success depended on whether 30, 80, 130 or 180 % of target size were defined as “hit area” (see Figure 1, upper part). The radius of the visible target was 15, 20, 25 or 30 px. There were three experimental blocks. Each block included 32 trials. In each block, each combination of target size and feedback factor was presented twice in a randomized order. After a block the achieved number of hits was reset. The participants were encouraged to increase the hit rate and to improve the quality of judgments in the next block. At the beginning of the experiment participants performed eight practice trials, which were not included in the analyses. The general instruction provided to the participants at the beginning of the experiment stressed accuracy of movements as well as of the judgments.

#### Data preprocessing

Trials in which movement distance (distance between start position and the endpoint of the pointing movement along the Y-axis) was less than 160 px were excluded. Subsequently, trials in which estimated radii, movement times or movement distances were below or above 2 *SD* of the mean as computed for each participant, each target, and each feedback factor were also excluded. Overall, 97.7% of trials entered the analyses.

### Results

The hit rates (i.e., performance with respect to the manipulated feedback) increased with an increase in target size and with an increase in feedback factor as expected (see Table 1). An ANOVA with target size and feedback factor as within-subjects factors revealed significant main effects for both factors, *F*_(3, 63)_ = 52.0, *p* < 0.001, η^2^_*p*_ = 0.712, and *F*_(3, 63)_ = 181.0, *p* < 0.001, η^2^_*p*_ = 0.896. Additionally, a significant interaction was observed, *F*_(9, 189)_ = 2.1, *p* = 0.031, η^2^_*p*_ = 0.091, which was mainly due to a smaller increase of hit rates with target size for the smallest feedback factor.

**Table 1:** **Mean feedback related hit rates [%] according to target size and feedback factor in Experiment 1**.

**Target radius [px]**		**15**	**20**	**25**	**30**
Feedback factor	0.3	7.0 (10.0)	6.1 (8.2)	13.2 (13.7)	16.6 (21.0)
	0.8	28.2 (21.6)	34.8 (25.7)	52.7 (33.2)	56.8 (35.1)
	1.3	48.5 (28.1)	64.4 (29.2)	69.5 (28.9)	82.4 (27.5)
	1.8	67.3 (29.7)	77.1 (25.4)	90.0 (14.3)	93.0 (14.4)

Standard deviations are shown in parentheses.

An ANOVA including “real” hit rates (i.e., performance with respect to the visible target size) yielded a significant main effect of target size, *F*_(3, 63)_ = 46.5, *p* < 0.001, η^2^_*p*_ = 0.689 (all other *p*s > 0.3). An increase in target size was associated with an increase in real motor performance (see Table 2).

**Table 2:** **Mean hit rates [%] relating to the visible target surface in Experiment 1**.

**Target radius [px]**		**15**	**20**	**25**	**30**
Feedback factor	0.3	39.5 (27.2)	46.8 (26.8)	61.1 (26.2)	70.9 (27.6)
	0.8	35.9 (28.4)	47.7 (34.2)	60.6 (37.0)	66.1 (32.8)
	1.3	37.9 (26.3)	46.1 (29.3)	61.2 (32.6)	67.3 (30.1)
	1.8	35.9 (25.4)	53.2 (33.9)	65.0 (24.2)	70.9 (29.2)

Standard deviations are shown in parentheses.

**Figure 2:**
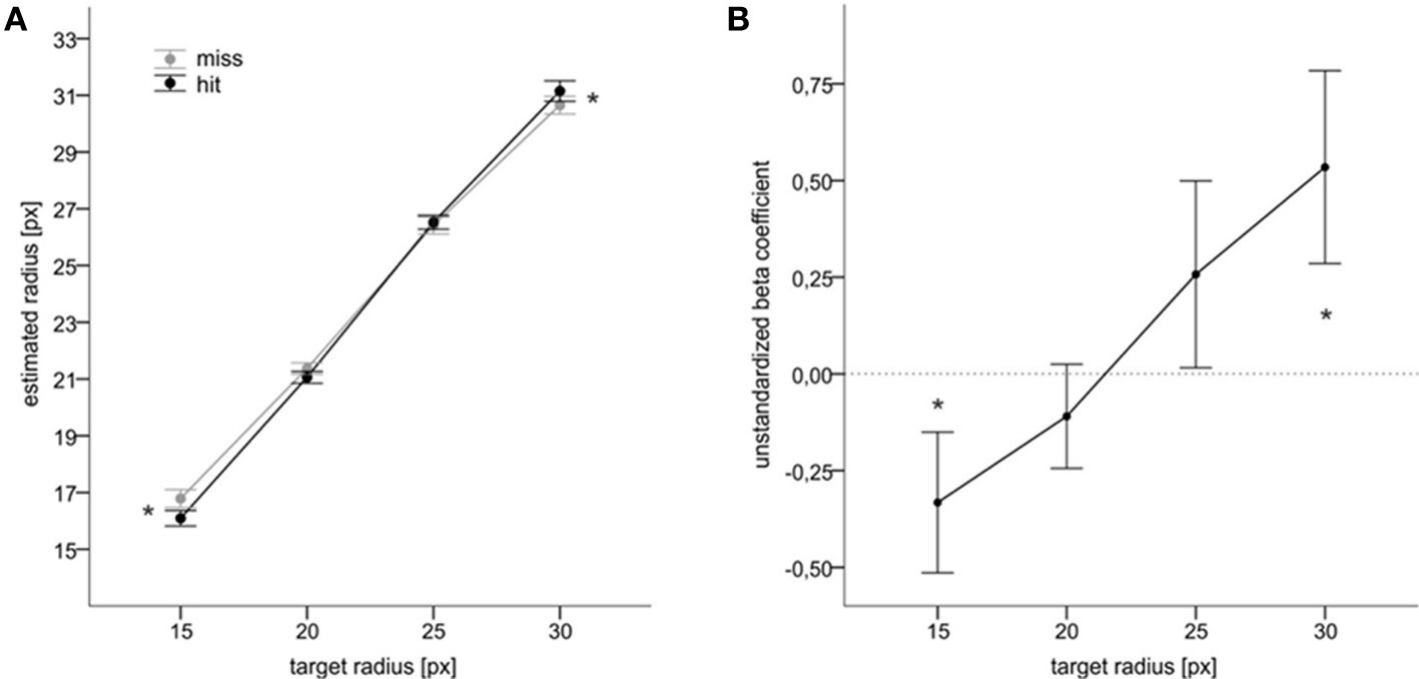
**Main results of Experiment 1. (A)** Mean judgments of target radius as a function of target size and of action success. **(B)** Mean unstandardized beta coefficients indicating an impact of feedback on size judgments for each target condition (cf. text). Error bars reflect standard errors. Asterisks denote statistical significance (*p* ≤ 0.05).

We then analyzed the judgments of target size depending on whether a hit or miss was fed back (feedback of result). We computed means of estimated target size for each participant, target size (15, 20, 25, 30) and category of performance feedback (hit vs. miss), and subjected these mean estimates to an ANOVA with target size (15, 20, 25, 30) and performance feedback (hit vs. miss) as within-subjects factors. This ANOVA revealed a significant main effect of target size, *F*_(3, 63)_ = 892.3, *p* < 0.001, η^2^_*p*_ = 0.977, and a significant interaction between target size and performance feedback, *F*_(3, 63)_ = 6.4, *p* = 0.001, η^2^_*p*_ = 0.235. The estimates of target size increased with target size. Mean reproduced radii were 16.4, 21.2, 26.5, and 30.9 for target radii of 15, 20, 25, and 30 px respectively. More importantly, hits were associated with smaller estimates as compared to misses when the target size was rather small. For the two larger targets, this relationship was reversed. Figure 2A shows the according values. Follow-up analyses indicated significant differences between hits and misses for the smallest and for the largest targets with *t*_(21)_ = 3.1, *p* = 0.005 and *t*_(21)_ = 2.1, *p* = 0.050, respectively.

We also included feedback factor (4 levels: 0.3, 0.8, 1.3, and 1.8) instead of action success in an ANOVA of size judgments. This analysis revealed a marginally significant interaction between target size and feedback factor, *F*_(9, 189)_ = 1.8, *p* = 0.079, η^2^_*p*_ = 0.077 (additionally to a significant main effect of target size, see above). Size judgments tended to decrease with an increase in feedback factor for the smallest target and to increase for the largest target (see Table 3 for means). If only these two target conditions were included, a target size *x* feedback factor interaction was significant, *F*_(3, 63)_ = 3.8, *p* = 0.014, η^2^_*p*_ = 0.154. That is, the varied probability to achieve a hit affected judgments differently depending on target size in a similar way as in the previous analysis (i.e., judgments tended to decrease/increase with an increase in (informed) hitting performance for the smallest/largest target).

**Table 3:** **Mean judgments of target radius as a function of target size and of feedback factor**.

**Target radius [px]**		**15**	**20**	**25**	**30**
Feedback factor	0.3	16.95 (1.9)	21.02 (1.3)	26.23 (1.6)	30.75 (1.8)
	0.8	16.61 (1.7)	21.33 (1.0)	26.81 (1.4)	31.12 (1.6)
	1.3	16.34 (1.1)	21.38 (1.2)	26.28 (1.4)	31.34 (1.8)
	1.8	16.28 (1.3)	21.03 (1.1)	26.50 (1.5)	30.93 (1.6)

Standard deviations are shown in parentheses.

These first analyses suggest that action feedback (hit or miss) has an impact on judgment of target size. In a second analysis we tested whether this impact still holds after possible influences of actual movement performance on perceived target size have been analytically removed^3^.

For this purpose we initially performed a multiple regression including the spatial deviation of movement endpoints in each trial from the center of the target as a predictor and reproduced radius of the target as the criterion. This analysis was done for each target size and each subject separately, and aimed to remove all the variance in target judgments which may be due to motor variability. This initial analysis revealed positive regression coefficients (unstandardized) for each target on average (0.01, 0.03, 0.01, and 0.01 for target radii 15, 20, 25, and 30 respectively) delineating larger size estimates with an increase in movement deviation from the center of the target. However, none of the mean coefficients was significantly different from zero for the whole sample of participants (two-tailed *t*-tests: *p* = 0.503, *p* = 0.063, *p* = 0.493, and *p* = 0.319) indicating no substantial impact of actual motor performance on perceptual estimates in the present data.

In the second step, we then examined whether the presented feedback of motor performance explains some of the residual variance. That is, we subjected the residual size estimates (i.e., observed judgment of target size minus predicted judgment of target size based on movement deviation) to another regression analysis. In this second analysis, presented movement feedback served as the predictor (the value of 0 was assigned for misses and the values of 1 for hits) and the residuals from the first step were used as the criterion.

Figure 2B illustrates mean unstandardized beta coefficients from this analysis. The beta coefficients significantly increased with an increase in target size, *F*_(3, 63)_ = 3.4, *p* = 0.023, η^2^_*p*_ = 0.139. Moreover, the coefficients were significantly larger than zero for the largest target, *t*_(21)_ = 2.1, *p* = 0.022, and significantly smaller than zero for the smallest target, *t*_(21)_ = 1.8, *p* = 0.041 (one-tailed^4^). This pattern of results reassured the results observed with the analysis based on aggregated data (i.e., ANOVA of mean values) and indicated that independently of motor performance participants tended to judge the small target as larger and the large target as smaller after the feedback of a miss than after the feedback of a hit.

### Discussion

The main finding of Experiment 1 was a tendency of participants to judge relatively small targets as larger and relatively large targets as smaller after misses than after hits. This difference in estimates after successful vs. failed pointing movements was especially evident for the smallest and the largest targets and it decreased for the intermediate target sizes. Moreover, this pattern was independent of real motor performance indicating an impact of action evaluation processes. In other words, participants showed a bias toward the middle of the target range during judgments of target size after apparent target misses as compared to trials in which the target was apparently hit. These results indicate that perceptual estimates measured after an action may not exclusively reflect actor's action abilities but may also be the result of action evaluation processes.

## Experiment 2

The main purpose of Experiment 2 was twofold. First, given a rather complex outcome of Experiment 1, we asked whether the results were conceptually replicable. Second, we were also interested in the effect of the Ebbinghaus Illusion on motor behavior reported by Witt et al. (2012). These authors reported increased sport performance in a putting task when smaller context stimuli surrounded a hole as compared with larger context stimuli.

**Figure 3:**
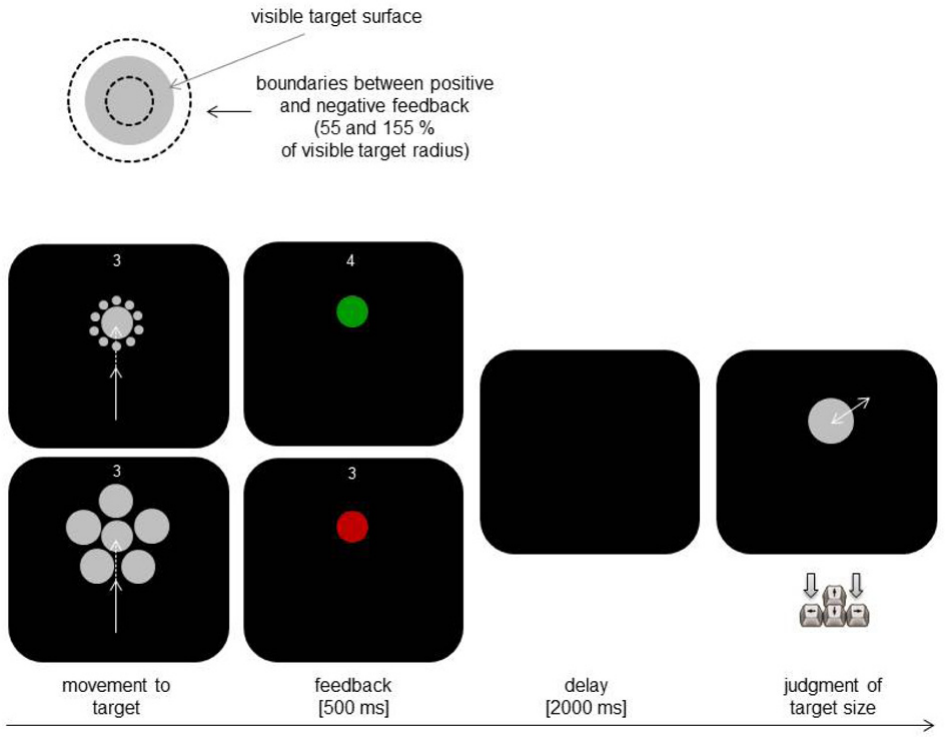
**Schematic illustration of the critical manipulation (upper part) and of the main trial events in Experiment 2 (lower part)**.

The main task and the main procedure were the same as in Experiment 1. The participants made repetitive movements aiming at hitting a target and judged target size after each movement. The implemented changes were related to the number of used targets, the areas serving for positive feedback, and to target context. We now used two targets and two feedback conditions (instead of four). Additionally, each target was surrounded by small or large context circles serving to induce the Ebbinghaus Illusion (cf. Figure 3).

According to the results of Experiment 1 we expected to find an interaction between target size and action success, i.e., a decrease in size estimates after hits than after misses for the smaller target and a reversed pattern for the larger target. Moreover, based on the results of Witt et al. (2012) we predicted a decrease in error rates for small context stimuli as compared with larger context stimuli. Additionally, the present setup allowed us to test whether the used method of adjustment is suitable to measure changes in apparent target size. If so then small context stimuli should increase the reported target size as compared with large context stimuli (i.e., the usual Ebbinghaus illusion should be observed).

### Methods

#### Participants

Twenty-four participants participated. They gave informed consent for the procedures, and received monetary compensation or course credit in exchange. The hitting rate of one participant was very low (about 5%). His data was excluded from analyses. The final sample included 17 females and 6 males. The mean age was 28 years ranging from 19 to 55 years of age (*SD* = 7). All of them were right handers.

#### Procedure and design

The procedure and the design of Experiment 2 were very similar to those of Experiment 1 with few exceptions. In Experiment 2, we used only two targets with radii of 20 and of 25 px (instead of four). Moreover, during the pointing movements the targets were surrounded by additional gray circles. There were two target context conditions. In one of them, ten context circles were 5 px in size and were either 30 px (smaller target) or 35 px (larger target) away from the target (center to center). In another condition, five context circles were 35 px in size and were either 60 or 65 px away from the target. We also used only two feedback factors instead of four with 1:0.55 and 1:1.55 relations of target radius to the radius of hit area.

#### Data preprocessing

Data was preprocessed in an analogous way as in Experiment 1. Trials with movement distances less than 160 px were discarded. Also, trials in which estimated radii, movement times, or movement amplitudes were below or above 2 *SD* of the mean (computed for each participant, each target, each target context, and each feedback factor) were also excluded. Overall, 91.5% of trials entered the analyses.

### Results

The feedback related hit rates varied in a predicted direction as a function of target size and feedback factor, *F*_(1, 22)_ = 13.0, *p* = 0.002, η^2^_*p*_ = 0.372, *F*_(1, 22)_ = 278.3, *p* < 0.001, η^2^_*p*_ = 0.927. An effect of visual illusion on these hit rates, however, was not observed (all *p*s > 0.2). An overview of mean values is given in Table 4.

**Table 4:** **Mean feedback related hit rates [%] according to target size, target context and feedback factor in Experiment 2**.

**Target radius [px]**		**20**		**25**	
**Context stimuli**		**Small**	**Large**	**Small**	**Large**
Feedback factor	0.55	20.5 (21.7)	25.0 (17.7)	35.3 (21.7)	32.8 (24.1)
	1.55	76.6 (29.3)	80.5 (19.8)	84.6 (24.7)	84.1 (21.0)

Standard deviations are shown in parentheses.

An ANOVA including hit rates relating to the “real” performance (i.e., with respect to the visible target size) yielded a significant main effect of target size, *F*_(1, 22)_ = 31.2, *p* < 0.001, η^2^_*p*_ = 0.586. Additionally, significant interactions were observed between target size and target context, *F*_(1, 22)_ = 4.7, *p* = 0.042, η^2^_*p*_ = 0.175, and between target context and feedback factor, *F*_(1, 22)_ = 7.1, *p* = 0.014, η^2^_*p*_ = 0.245. Also, the three-way interaction was marginally significant, *F*_(1, 22)_ = 4.0, *p* = 0.058, η^2^_*p*_ = 0.153. As in Experiment 1, an increase in target size was also associated with an increase in real motor performance. Additionally, for each target size, performance varied to some extent depending on target context and feedback factor (see Table 5 for means). Importantly, however, in no condition performance systematically increased when the target was surrounded by small context stimuli as compared with large context stimuli (*p* > 0.5). For the small target and the small feedback factor, hit rates even decreased when the context included small stimuli, *t*_(22)_ = 3.7, *p* < 0.05.

**Table 5:** **Mean hit rates [%] relating to the visible target surface in Experiment 2**.

**Target radius [px]**		**20**		**25**	
**Context stimuli**		**Small**	**Large**	**Small**	**Large**
Feedback factor	0.55	43.0 (28.6)	63.3 (26.3)	68.3 (27.6)	67.5 (27.3)
	1.55	53.6 (32.2)	53.6 (23.6)	65.7 (26.9)	63.4 (25.5)

Standard deviations are shown in parentheses.

For each subject, perceptual estimates were averaged for each target size, each target context, and each performance feedback condition. One missing value (small target, small context, hit) was replaced by the mean of the sample in the corresponding condition. An ANOVA with target size, target context, and performance feedback as factors revealed significant main effects for target size and target context with *F*_(1, 22)_ = 539.6, η^2^_*p*_ = 0.961 and *F*_(1, 22)_ = 65.9, η^2^_*p*_ = 0.750 respectively. Additionally to the trivial effect of target size, this result indicated the expected impact of visual illusion: the target was judged larger when it was surrounded by small context stimuli (*M* = 25.3 px) than when it was surrounded by large context stimuli (*M* = 23.1 px).

**Figure 4:**
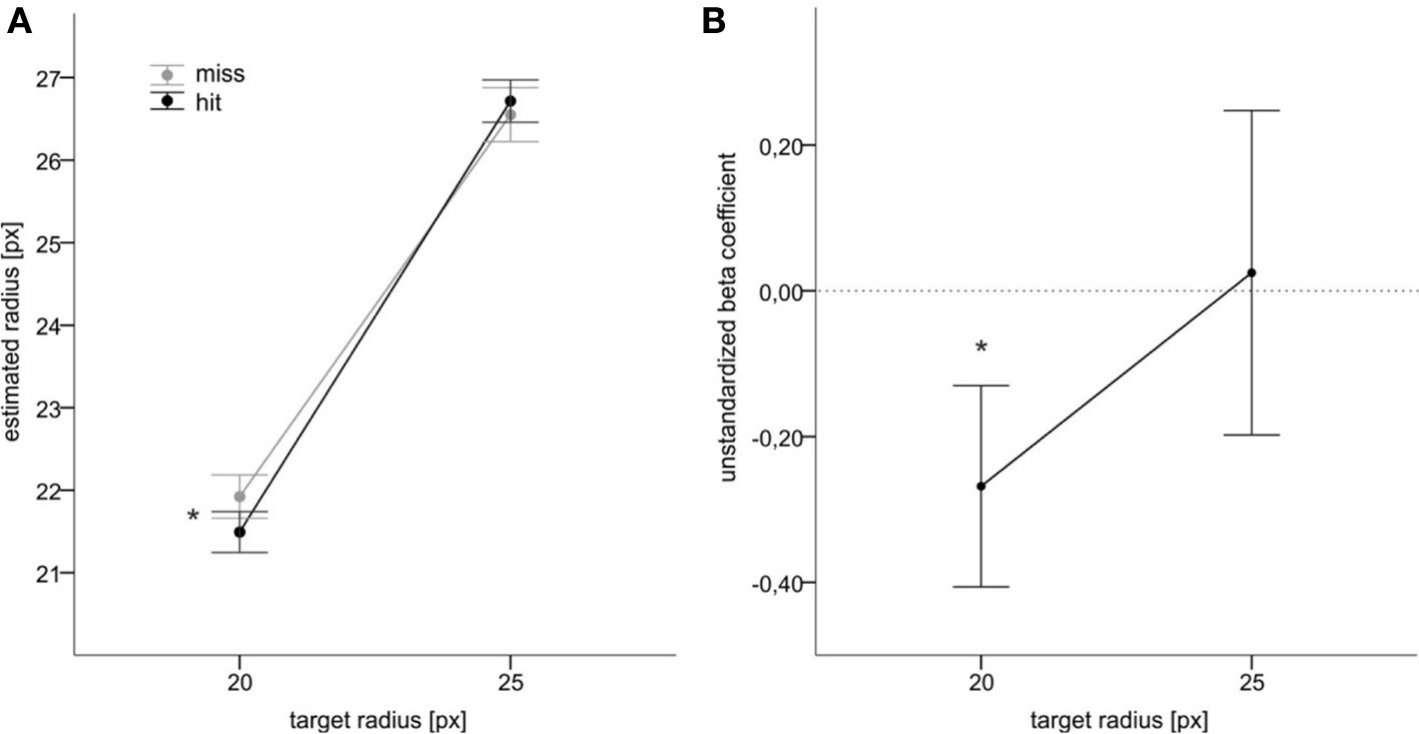
**Main results of Experiment 2. (A)** Mean judgments of target radius as a function of target size and of action success. **(B)** Mean unstandardized beta coefficients for both targets. Error bars reflect standard errors. Asterisks denote statistical significance (*p* ≤ 0.05).

Importantly, an interaction between target size and performance feedback was also significant, *F*_(1, 22)_ = 6.1, *p* = 0.022, η^2^_*p*_ = 0.216. As shown in Figure 4A, when the target was small, there was a decrease in judgments for hits as compared to misses, *F*_(1, 22)_ = 6.6, *p* = 0.018, η^2^_*p*_ = 0.231. For the larger target, in contrast, there was no significant difference between both feedback conditions, *F*_(1, 22)_ = 0.6, *p* = 0.4, η^2^_*p*_ = 0.019.

We again performed a two-step regression analysis (cf. Experiment 1) to control for motor influences on size estimations. This analysis was performed for each participant and each target separately. The first analysis, in which the spatial deviation of movement endpoints from the center of the target served as the predictor and reproduced radius of the target was used as the criterion revealed mean unstandardized beta values of.02 (*p* = 0.296) and of −0.002 (*p* = 0.830) for the smaller and larger targets, respectively. Similarly to the results of Experiment 1, this result did not indicate systematic influences of the actual motor performance on judgments of target size across participants.

The mean unstandardized regression coefficients from the second analysis are shown in Figure 4B. For the smaller target, the mean coefficient's deviation from zero was significant, *t*_(22)_ = 1.9, *p* = 0.033, suggesting an influence of action feedback beyond motor performance.

### Discussion

The main result of Experiment 2 largely replicated the pattern of results observed in Experiment 1, namely a bias toward the middle of the target range during judgments of target size after misses as compared to judgments after hits. This bias, however, was only significant in the small target condition.

The results also revealed a significant effect of the implemented Ebbinghaus illusion on judgment behavior: small context stimuli were associated with larger size judgments than with large context stimuli. This indicates that the used method of adjustment is suitable to measure changes in apparent target size. This also suggests that changes in judgment behavior following feedback manipulations were due to subjective changes in perceived target size rather than due to other task specific factors.

Motor behavior, as expressed in hit rates, was not susceptible to the Ebbinghaus Illusion as expected (i.e, neither actual nor explicitly informed hit rates increased when small context stimuli surrounded the target as compared with large context stimuli). Thus, the results of Witt et al. (2012) were not replicated. We assume that the implemented manipulation of performance feedback might have been responsible for the lack of this effect. Targets that appear bigger may signal that they are easier to hit (cf. Witt et al., 2012), but because this expectancy was often violated in the present study, an adjustment of motor behavior did not occur. This would imply that adjustments of motor behavior are modulated by expectancies of goal achievement (cf. Witt et al., 2012).

## Experiment 3

**Figure 5:**
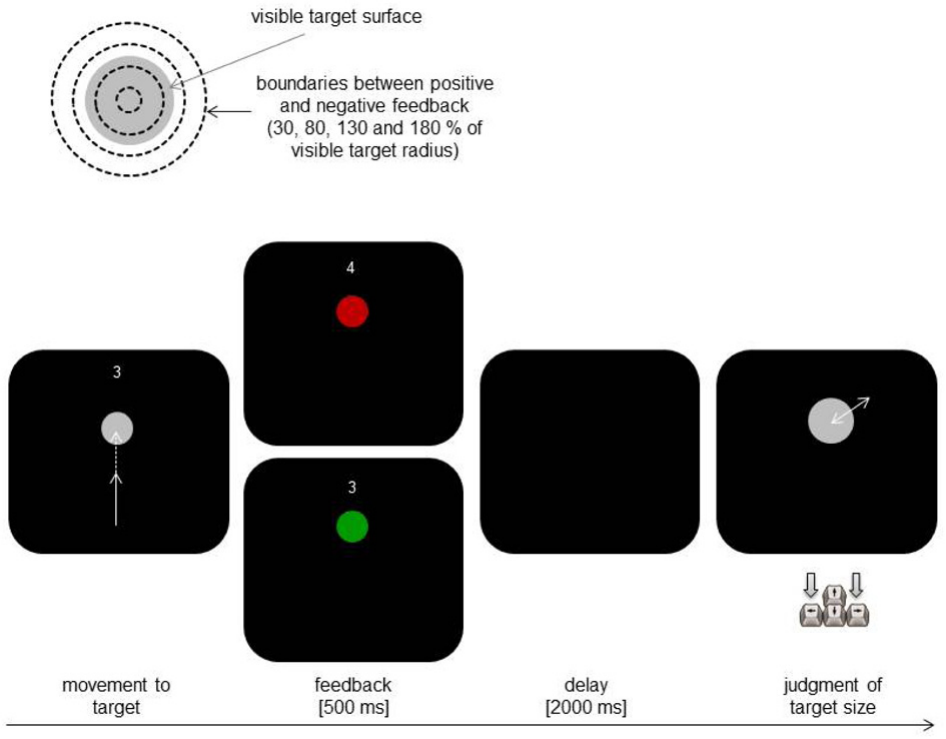
**Schematic illustration of the critical manipulation (upper part) and of the main trial events in Experiment 3 (lower part)**.

One possible caveat of Experiments 1 and 2 was that the color of the target during the feedback phase might have had an effect on the size estimates rather than the experience of success or failure. To examine whether this confounding factor may explain the observed results was the primary goal of Experiment 3. Accordingly, we interchanged the colors of the target presented during the feedback of movement outcome. In particular, targets turned from gray to red after hits and from gray to green after misses (cf. Figure 5). If the observed bias toward the middle of the target range after misses is due to the feedback color then the effect observed in Experiment 1 should be reversed. If however, an experience of success vs. failure is the crucial aspect then results similar to those of Experiment 1 are expected.

### Methods

#### Participants

Twenty-four participants participated. They gave informed consent for the procedures, and received monetary compensation or course credit for their participation. One participant hit the targets only rarely (in about 2% of trials). Her data was excluded from analyses. The final sample included 20 females and 3 males. The mean age was 28 years ranging from 19 to 54 years of age (*SD* = 7). All of them reported to be right handers.

#### Procedure and design

Experiment 3 was almost identical to Experiment 1. The critical difference was related to the color of the target during the performance feedback. Red color signaled a hit now and green color signaled a miss (cf. Figure 5). We also replaced the size of two targets (see also Footnote 1) and used target radii of 16, 20, 26, and 30 px.

#### Data preprocessing

Data preprocessing was performed in an analogous way as in Experiment 1. Overall, 98.0% of trials entered the analyses.

### Results

As in Experiment 1 and 2, feedback related hit rates increased with target size and with an increase in feedback factor, *F*_(3, 66)_ = 59.6, *p* < 0.001, η^2^_*p*_ = 0.730, and *F*_(3, 66)_ = 212.4, *p* < 0.001, η^2^_*p*_ = 0.906. Also, the target size x feedback factor interaction was also significant, *F*_(9, 198)_ = 2.7, *p* = 0.005, η^2^_*p*_ = 0.109, indicating a smaller increase in hit rates with target size for the smallest feedback factor. Table 6 shows the mean values from this analysis.

**Table 6:** **Mean feedback related hit rates [%] according to target size and feedback factor in Experiment 3**.

**Target radius [px]**		**16**	**20**	**26**	**30**
Feedback factor	0.3	4.1 (9.9)	6.5 (10.9)	11.7 (11.8)	14.8 (19.1)
	0.8	33.2 (25.2)	40.1 (25.1)	63.8 (28.5)	59.6 (25.9)
	1.3	57.5 (33.5)	69.6 (30.0)	79.3 (22.5)	86.2 (19.9)
	1.8	74.6 (21.8)	87.4 (17.3)	92.8 (9.8)	95.4 (10.0)

Standard deviations are shown in parentheses.

When motor performance with respect to the visible target size was included in the analysis a significant main effect of target size, *F*_(3, 66)_ = 52.2, *p* < 0.001, η^2^_*p*_ = 0.704, was observed (other *p* > 0.2). An increase in target size was again associated with an increase in real motor performance (see Table 7 for means).

**Table 7:** **Mean hit rates [%] relating to the visible target surface in Experiment 3**.

**Target radius [px]**		**16**	**20**	**26**	**30**
Feedback factor	0.3	46.4 (33.7)	52.6 (22.1)	70.0 (24.0)	73.0 (24.8)
	0.8	47.5 (28.0)	54.1 (24.8)	75.7 (27.1)	74.2 (19.5)
	1.3	40.7 (29.5)	54.6 (28.3)	67.8 (27.8)	74.6 (24.6)
	1.8	43.5 (29.6)	58.0 (25.4)	64.2 (24.8)	79.7 (20.0)

Standard deviations are shown in parentheses.

**Figure 6:**
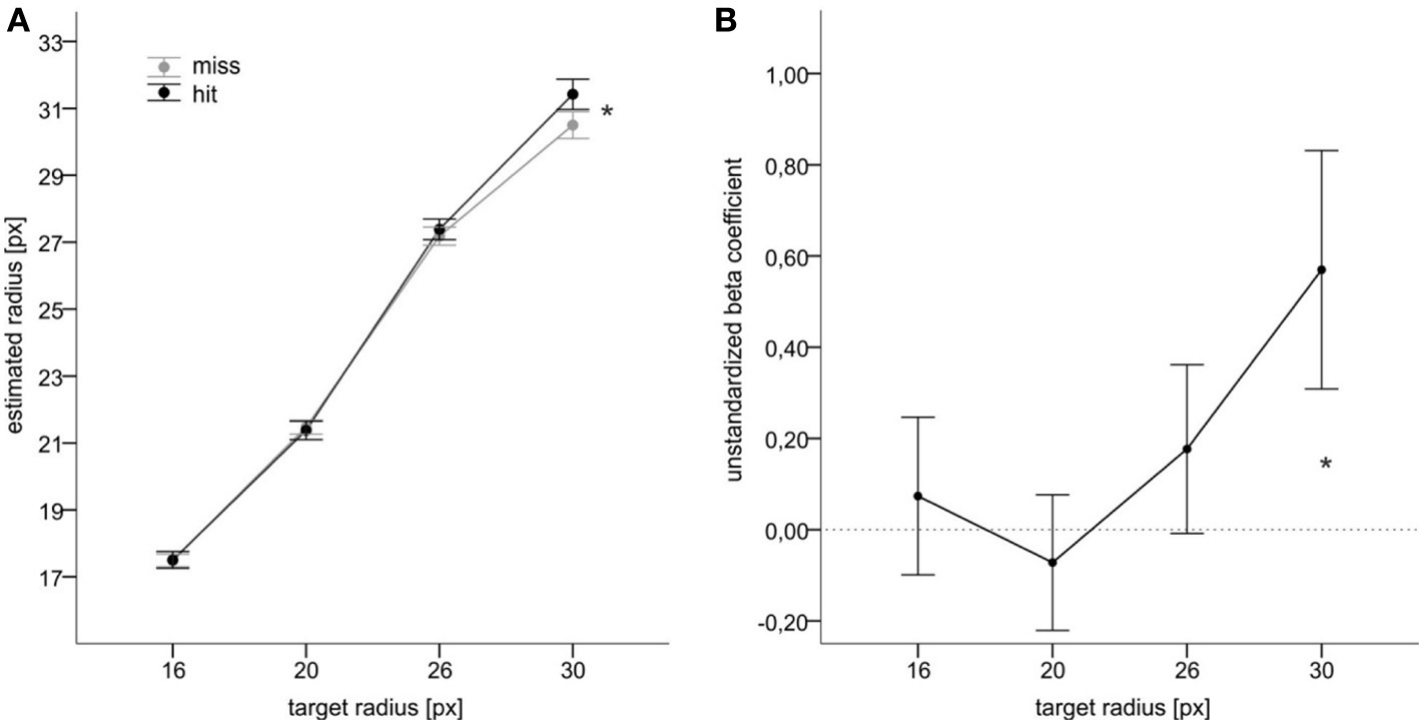
**Main results of Experiment 3. (A)** Mean judgments of target radius as a function of target size and of action success. **(B)** Mean unstandardized beta coefficients for each target condition. Error bars reflect standard errors. Asterisks denote statistical significance (*p* ≤ 0.05).

As in Experiment 1, perceptual estimates were averaged for target sizes and performance feedback conditions. An ANOVA with target size and performance feedback as factors revealed a significant main effect for target size, *F*_(3, 66)_ = 1162.3, *p* < 0.001, η^2^_*p*_ = 0.981, and a significant interaction between performance feedback and target size, *F*_(3, 66)_ = 4.6, *p* = 0.006, η^2^_*p*_ = 0.173. The observed interaction suggested that for the largest target stimuli there was a bias to judge the target as larger after a hit than after a miss, *t*_(22)_ = 2.8, *p* = 0.006. For the other target sizes there were no significant differences between hits and misses (all *p*s > 0.182). Figure 6A illustrates the mean values from these analyses.

The results of the first regression analysis testing for the impact of actual motor performance on size judgments (cf. Experiment 1 and 2) yielded unstandardized beta values of 0.0005 (*p* = 0.960), 0.005 (*p* = 0.532), −0.02 (*p* = 0.098), and −0.05 (*p* = 0.022) for the target radii 16, 20, 26, and 30 px respectively. This result indicated a bias to judge the two larger targets as larger the closer the endpoint of the movement was to the center of the target.

The main results of the second regression are shown in Figure 6B. The unstandardized beta coefficients tended to increase with an increase in target size as in Experiment 1. A main effect of target size was marginally significant, *F*_(3, 66)_ = 2.4, *p* = 0.074, η^2^_*p*_ = 0.099. The mean coefficient was significantly different from zero only for the largest target, *t*_(22)_ = 2.2, *p* = 0.020. These results confirm the ANOVA results and indicate that the observed bias of judging the largest target as smaller following feedback of a miss than of a hit is rather independent of whether participants really hit or missed the target with their movement.

### Discussion

Despite the change in the targets' feedback color, a similar interaction of target size and performance feedback as in Experiments 1 and 2 was observed. Thus, the main pattern of results was not reversed and thus, does not depend on the color of the target during performance feedback. However, unlike in Experiment 1 no effect of performance feedback was observed for the smaller targets. Perhaps, this rather unusual assignment of colors to success conditions (red is typically associated with errors) made it more difficult to attribute an outcome to success or failure in case of small targets than in case of larger targets, which are generally associated with a stronger sensory stimulation. As a consequence, after movements toward smaller targets participants may have experienced larger uncertainty whether a given movement was successful or not.

## General discussion

The main purpose of the present study was to examine the relation between feedback about action success and perceived size of action-related objects. Participants made pointing movements aiming to hit a circular target of varying size and judged target's size after each movement. Via manipulating the performance feedback we tried to disentangle the impact of action ability from the effect of experienced action success (i.e., of knowledge of results). We hypothesized that the experience of success and failure modulates perceptual judgments of target size independently of actual action ability. The present results support this hypothesis. In three experiments, a significant interaction between target size and fed back action success (hits vs. misses) was observed. In Experiment 1, a small target was judged to be larger and a large target to be smaller after misses than after hits. In Experiment 2, a small target was again judged to be larger after misses, but there was no significant impact of action success on estimations of the larger target. In Experiment 3, in contrast, a larger target was judged to be smaller after misses than after hits, but no significant differences between hits and misses for smaller targets. Despite some inconsistency, the whole pattern of results indicates that participants tended toward the middle of the used target range in their size estimations, after they experienced a failure of their motor performance. This bias proved to be independent from real motor behavior suggesting its rather non-motor origin.

Related phenomena are well known since the beginning of the last century: the estimates of individual stimuli often shift toward the central value of the presented set of stimuli in different modalities and in diverse context conditions (Hollingworth, 1910). This *central tendency effect* has been assumed to reflect the participants' tendency to evaluate the physical value of a current stimulus on the basis of an internal reference including previously and actually experienced stimuli (e.g., Helson, 1964). There is evidence suggesting that the emergence and the magnitude of such range effects may crucially depend on the quality of stimulus information. Elfering and Sarris (2006), e.g., observed an increase of a stimulus range effect with an increase in delay and with a decrease in presentation time in a memory task. Cicchini et al. (2012) reported a usual central tendency to the mean in non-musicians in an interval timing reproduction task, whereas expert drummers did not show such a bias. In a related task Jazayeri and Shadlen (2010) demonstrated a strong dependence of the magnitude of the central tendency effect on the magnitude of the sample intervals: the longer the sample intervals the stronger was the effect. Thus, with an increase in uncertainty (or in ambiguity of stimulus information), participants seem to apply the central-tendency strategy increasingly. It has been argued that such behavior reflects a kind of compensation mechanism serving to optimize the overall performance (Jazayeri and Shadlen, 2010; Cicchini et al., 2012).

A related mechanism may also account for the present results. Experiencing a non-successful action may induce some uncertainty about the size of the seen target (possibly due to a violation of motor predictions). As a consequence, participants may try to reduce this ambiguity by applying more weight to an average size of the target range compared to when they experienced a successful action (for which motor predictions are confirmed by feedback of action outcome). In other words, participants might stronger rely on their memory of previous events after misses than after hits.

The present results do, of course, not rule out that action ability might modulate perception in related setups independently of action evaluation processes. Lee et al. (2012), e.g., found a systematic positive relation between action accuracy and judgments of target size in an archery context in which participants did not have explicit knowledge of the success of their motor performance. In the present experiments, we found only little evidence for a systematic impact of actual motor performance on judgments of target size: only in Experiment 3 actual motor performance significantly predicted estimated target size for one of the used targets in a previously often observed manner (the better the performance the larger the estimate). This outcome, however, might be not surprising due to the nature of the present task in which actual and fed back performance may diverge. As a consequence of such an experienced mismatch, the participants might not have seen the need to change their view on the target depending on their actual ability. For instance, perceiving a target as smaller after (or before) a miss may help to adjust future behavior (i.e., to exert more resources) as mentioned earlier (Witt and Dorsch, 2009). Such a strategy, however, would not be very effective in the present experiments, in which an increase in effort may even be associated with a worse (fed back) performance.

It should also be noted that the task used in the present study differs in some possibly important ways from comparable but ecologically more valid settings in sports. Typically, knowledge of result is not independent from action ability. Accordingly, external feedback about action success experienced by the actor is often in accordance with the internal movement feedback (provided, e.g., by the kinesthesia). The implemented decoupling of feedback from the actual motor performance in the present task inevitably resulted in a rather artificial situation in which this innate relation (between ability and knowledge of results) was disrupted. Thus, the present results and drawn conclusions might be limited to comparable situations. In other words, action evaluation processes may affect perception primarily if action outcome deviates from possible predictions derived from body perception. This points to another important issue which is not well understood at present. Long-term developed expectancies from top experts which are based on former experience of action performance and action success might have an impact on size estimations of action relevant objects in addition to the current ability and evaluation processes.

Moreover, in many sports such as in golf, darts, or basketball, bodily movements substantially determining the success of an action do not last until action outcome is known. Accordingly, when the actor receives external feedback about whether his action was successful or not s/he is not any longer able to verify this information with a current internal source. In the present task, however, this possibility existed and this caveat may possibly limit the range of validity of results to some extent.

To conclude, our results indicate that caution is needed in drawing conclusions from perceptual estimates measured after an action outcome is known because this data might include influences from variables which are not directly related to certain motor factors such as “ability.”

## Author note

This research was supported by grant KI 1620/1-1 awarded to Wladimir Kirsch by the German Research Council (DFG). The publication was funded by the DFG and the University of Wuerzburg within the funding program Open Access Publishing.

### Conflict of interest statement

The authors declare that the research was conducted in the absence of any commercial or financial relationships that could be construed as a potential conflict of interest.

## Footnotes

^1^Separating influences of action ability from evaluation of action outcome might seem like hair-splitting given that both factors are highly dependent in real life tasks (like in sports). However, whether the reported perceptual distortions are the results of processes directly linked to action ability (e.g., to body-related feedback mechanisms informing the actor about forthcoming or achieved action success) or rather to cognitive processes relating to evaluation of action outcome is an important theoretical question. A successful golfer, e.g., may judge the hole as larger just because s/he reasons “I was hitting the hole so often it should thus be quite huge” (cf. Cooper et al., 2012). If so, then his judgment behavior would be critically determined by processes associated with outcome evaluation rather than by his actual hitting ability.

^2^As a consequence of this procedure start radii and (the reproduced radii) related to uneven target radii were not integers. This implied the possibility that a possible modulation of estimates related to uneven targets might have been due to a bias in pressing a button that is not necessarily accompanied by changes in visible surface of the reproduced target. This, however, did not appear to be the case: across the experiments the critical effect was present for even as well as for uneven targets.

^3^One may suggest to further split the data not only depending on whether a hit or miss was fed back (as done in the analysis above) but additionally on whether the target was actually hit or missed. This approach has two disadvantages, however, First, not all combinations of the ensuing independent variables (actual performance, fed back performance, target size) occurred with each participant. In other words, we would face a loss of participants and a corresponding drop of power. Second, whereas the fed back performance is a discrete variable (hit or miss) the actual performance (spatial deviation from target center) is a metric variable. Transforming this metric variable to a discrete one (by dichotomizing it into cases in which spatial deviation was smaller or larger than target radius) would mean another loss of information and statistical power. An approach that elegantly circumvents these problems was recently proposed by Notebaert and Verguts (2007). The idea is to predict the relevant dependent variable by all available predictor variables that are not of current interest in a first step. This is done by running a multiple regression for each individual participant (hence, trial is the level of analysis here). One advantage is that discrete and metric independent variables can be used equivalently. In a second step it is then tested whether the residuals from that analysis (hence the variance of the dependent variable not already explained by the independent variables of the first step) can be predicted by the independent variable of interest.

^4^Here we used one-tailed *t*-tests due to predicted directions of the effects derived from the ANOVA results.
